# Emergent Pragmatic Conventions in Spoken ELF Corpus Data: Micro-Diachronic Analysis of Inclusive vs. Exclusive Multilingual Practices

**DOI:** 10.1007/s41701-024-00166-1

**Published:** 2024-05-30

**Authors:** Marie-Luise Pitzl-Hagin

**Affiliations:** Johannes Kepler University Linz, Linz, Austria

**Keywords:** English as a lingua franca, Pragmatic conventions, Multilingual practices, Transient International Groups, Micro-diachronic analysis of interaction, Spoken corpora

## Abstract

This article examines multilingual practices as an example of emergent pragmatic conventions in three Transient International Groups (TIGs) using spoken English as a lingua franca (ELF) from the Vienna-Oxford International Corpus of English (VOICE). The analysis combines principles of corpus linguistics and conversation analysis by adopting a new approach for the micro-diachronic analysis of spoken interaction. Quantitative and qualitative evidence and micro-diachronic visualizations of VOICE transcripts show how the three groups examined interactively develop group-specific multilingual practices. The analysis reveals that the three groups have different preferences in this respect. While two groups develop inclusive multilingual practices in the course of their interaction, one group shows a tendency to use multilingual practices exclusively, primarily in side sequences. In addition to multilingual use, the presence or absence of metalinguistic discussions about language (and languages) plays a role for creation of shared transcultural territory and the formation of group identity. These processes are indicative of how unacquainted multilingual speakers negotiate and develop pragmatic conventions more generally.

## Introduction

Whenever groups of speakers meet for the first time, linguistic practices and pragmatic conventions cannot necessarily be taken for granted. Conventions might be adapted, negotiated and/or changed by means of and throughout interaction. This observation holds true for all situations in which interlocutors are unacquainted, but it is heightened when groups are linguistically and/or culturally heterogeneous. A prime example are interactions in which speakers use English as a lingua franca (ELF) as a common means of communication. In lingua franca contexts, interactants have multilingual repertoires that comprise situational Multilingual Resource Pools (MRPs, see Pitzl, 2016). Speakers’ multilingual repertoires and resulting MRPs in ELF encounters are thus more varied than in situations where interactants belong to the same speech community. As a consequence, pragmatic conventions in ELF (or other lingua franca) contexts are more diversified and malleable than in more homogenous first language (L1) contexts. ELF situations therefore provide us with prime opportunities to study how pragmatic conventions emerge, evolve and change during relatively short timespans (e.g. single conversations), especially when speakers are newly acquainted with each other.

Due to its inherent orientation towards multilingualism, ELF research has examined code-switching and multilingual practices for quite some time. Many existing studies (such as Klimpfinger, 2009 or Cogo, 2012) offer interesting insights into which elements of which *languages might be used for which purposes in different (E)LF situations.^1^ There has, however, been relatively little engagement so far with how particular multilingual practices actually come about in a group of speakers, i.e. how such practices are first initiated and subsequently negotiated in real time. Having access to empirical investigations of these processes is likely to contribute considerably not only to our understanding of ELF use and multilingual interaction, but can help us gain deeper insights into the emergence of group-specific pragmatic conventions more generally.

As data sources, spoken ELF corpora have a central role to play. Yet, research of this kind comes with methodological challenges, for instance concerning the nature of transcripts and availability of spoken mark-up and pragmatic annotation (or lack thereof). Another crucial methodological issue has to do with devising research protocols for data analysis that allow researchers to trace in detail how speakers negotiate pragmatic conventions over a fairly short period of time in interaction.

This article addresses these empirical and methodological challenges. It examines emergent multilingual practices in three different Transient International Groups (TIGs) using ELF data from the *Vienna-Oxford International Corpus of English* (*VOICE*). It combines corpus linguistic and conversation analytic principles by applying a new methodology for micro-diachronic analysis of spoken interaction. In doing so, it provides quantitative and qualitative evidence and micro-diachronic visualizations that show how the three TIGs develop inclusive and exclusive group-specific multilingual practices. The interactions analyzed are carried out in English as a lingua franca, but the TIGs framework and the methodology of the study could be used for research on other *languages—in particular for contexts in which speakers are linguistically and/or culturally diverse. Proposing a novel approach to the analysis of interactive spoken data, the paper thus contributes to the expansion of methods that can be seen as part of corpus pragmatics.

## ELF, Multilingual Practices and Emergent Pragmatic Conventions

For the past two decades, researchers have been describing characteristics of language use and interaction in situations in which English is used by speakers as a lingua franca (LF), i.e. as a common means of communication—often mixed with other *languages—among speakers with different L1 and/or regio-cultural backgrounds. ELF interactions are always inherently connected to multilingualism (see e.g. Seidlhofer, 2011; Jenkins, 2015; Hülmbauer, 2016; Cogo, 2018) and inter/transculturality (e.g. Zhu, 2015; Baker, 2022). They are very often contexts of transient language contact (Pitzl, 2016). The multilingual nature of ELF interactions is constituted most noticeably through speakers’ multilingual repertoires (see Busch, 2012), which are brought into contact with one another. This contact of individual multilingual repertoires results in a situational MRP that is unique to a particular group of (E)LF speakers (Pitzl, 2018). The availability of different multilingual resources in speaker’s repertoires and in the resulting situational MRP opens up the possibility for the occurrence of multilingual phenomena. These may be more or less overtly realized by speakers and may thus be more or less overtly observable in transcribed data.

Multilingual conversational phenomena are well-researched in ELF encounters, using a fairly wide spectrum of terminology. Terms and phenomena studied include code-switching (e.g. Klimpfinger, 2009; Pietikäinen, 2014; Brunner & Diemer, 2018), cognates (Hülmbauer, 2011), plurilingual resources (Franceschi, 2017), translanguaging (Cogo, 2020) and multilingual practices (Cogo, 2012; Konakahara & Tsuchiya, 2020). The holistic view on speakers’ multilingual repertoires and the multilingual resource pool adopted in this paper is compatible with a translanguaging (e.g. García & Li, 2014) and translingual (e.g. Canagarajah, 2013) perspective on language that is shared by many ELF scholars in recent years. This perspective

moves away from an emphasis on the L1 of speakers to the whole repertoire of sociolinguistic and cultural resources participants may bring into the exchange, which may include languages that participants have learnt or encountered in their lives, which they may know or use at different proficiency levels, but are available as resources together with their L1 in their repertoire (Cogo, 2018: 363).

As Cogo (2018) points out, this *translanguaging perspective* is to some extent different from a *code-switching perspective*, since the latter emphasizes borders and transitions between different *languages or examines the functions of other-*language elements in conversation. Nonetheless, “these two perspectives do not contradict each other [but] […] emphasise different aspects” (Cogo, 2018: 357). Following this position, both perspectives can be used productively in the same study.

In this sense, despite speakers’ multilingual repertoires being viewed holistically in this article (i.e. the translanguaging perspective), many instances of overtly multilingual elements in the data analyzed can be regarded as code-switches. Following Auer (1999: 310), code-switches are seen as “cases in which the juxtaposition of two codes (languages) is perceived and interpreted as a locally meaningful event by participants”. This juxtaposition of so-called ‘codes’ is especially relevant when elements from *languages other than *English are first used in an ELF encounter. Yet, the degree of ‘foreignness’ and potential noticeability of a non-*English word as a code-switch can change over time during an interaction.

In addition to multilingual elements being more (or less) overtly recognizable as non-*English or other-*language, such elements carry pragmatic significance, of course. They tend to fulfill functions like signaling (regio-)cultural identity or introducing a new idea (Klimpfinger, 2009: 360-366). They help speakers manage interpersonal relationships or project a personal or professional identity (Franceschi, 2017: 70-73). A central question with regard to the emergence of pragmatic conventions is whether and how initially norm-transcending code-switches become increasingly ‘normal’ for a group—to the point where they might become part of a group’s shared repertoire without being perceived as ‘foreign’ (see Kalocsai, 2014). Some groups might even be seen to engage in what Hazel (2017: 317) refers to as “langscaping […] where members explore and discover one another’s linguistic repertoires and social-geographic trajectories and histories” by mixing *languages more extensively as interaction progresses. As discussed in detail by Mortensen (2017), transient groups and communities will be particularly open to the negotiation of sociolinguistic—and also thus also pragmatic—norms, since their communicative practices will be situated rather low on a “scale of semiotic sedimentation” (Mortensen, 2017: 275).

Since the specific properties of (E)LF interactions are always determined by the particular context—what Hülmbauer (2011: 142) calls the “situationality factor”—groups will differ with regard to whether any pragmatic development from code-switching to translanguaging to group-specific multilingual practices is realized. Furthermore, groups are likely to be different with regard to how these processes manifest in interaction. Contextual parameters like the size of a group (i.e. the number of interactants) or the degree of linguistic diversity are likely to influence which instances of multilingual elements are used and how these are responded to by interlocutors. Furthermore, the local context of an interaction (e.g. city, country, region) may impact the use of multilingual elements (for instance by referring to local *languages or place names).

To make similarities and differences of emergent group-specific multilingual practices tangible, this article examines data from three groups in the *Vienna-Oxford International Corpus of English* (*VOICE*). Before the data are introduced in detail (Section "Data"), the next section addresses some general issues in corpus pragmatics that have to do with investigating emergent pragmatic conventions on the basis of corpus data.

## Investigating Emergent Pragmatic Conventions with Corpus Data: Combining Methodological Perspectives

Empirical work with corpora has certainly influenced research on pragmatics with many “[c]orpus pragmatic approaches typically adopt[ing] a quantitative perspective” (Jucker, 2018b: 455). Describing language use with corpora thus often means engaging with frequencies of occurrence. In the case of diachronic pragmatics, this involves tracing change (or specific changes) over periods of time. In tracing pragmatic change, quantitative methods often coincide with a focus on written language. This is not surprising since written corpora are numerous and often large in size. Studying pragmatic change in real-time interaction, however, requires access to analysis of interactive spoken data. Such data are time- and labor- and hence cost-intense and hence more difficult to obtain.

In consequence, spoken corpora tend to be fewer and smaller than written corpora, especially if naturally-occurring interactions are recorded and transcribed specifically for the purpose of building a corpus. With 500.000 words, the *London-Lund Corpus 2* (Põldvere et al., 2021), for instance, constitutes a respectable size for a spoken corpus. By comparison, the 11.5 million words of naturally-occurring conversations in the *Spoken BNC (British National Corpus) 2014* (see Love et al., 2017) comprise a rather large spoken corpus. Prominent ELF corpora like the *Vienna-Oxford International Corpus of English* (VOICE, analyzed in this paper), the corpus of *English as a lingua franca in academic settings* (ELFA) and the *Asian Corpus of English* (ACE) rank in the middle, with a size of one million words each.

If we consider the study of spoken language in interaction as a central area of pragmatics, this is traditionally quite distinct from corpus linguistics. In a volume on *Methods in pragmatics*, Jucker et al. (2018), for example, separate chapters on observational pragmatics (such as ethnography and conversation analysis) from chapters on corpus pragmatics. This can be seen as indicative of a methodological divide: While corpus pragmatics tends to be seen as predominantly quantitative (see also the editorial of this journal), studies on observational pragmatics are usually associated with “methods of analysis that are mainly qualitative and rely on relatively small sets of data, consisting, for instance, of transcriptions of audio- or video-recorded data” (Jucker, 2018a: 335). In consequence, the analysis of spoken interaction tends to go hand-in-hand with methodologies like conversation analysis (CA, e.g. Drew & Heritage, 1992), interactional sociolinguistics (e.g. Gumperz, 1999) and interactional linguistics (e.g. Couper-Kuhlen & Selting, 2018).

In contrast to other areas of linguistic inquiry, these two methodological strands—i.e. corpus linguistics and qualitative CA-related methodologies—overlap quite heavily in the study of ELF communication. This is, to some extent, a direct consequence of the nature of data typically of interest for descriptive work on ELF. When ELF research started out in the late 1990’s, scholars like Firth (1996), House (1999) and Jenkins (2000) analyzed individual (non-public) interactive data sets. Shortly afterwards ELF corpus building began to take shape in Austria (*VOICE*) and Finland (*ELFA*). For these corpus building projects, a corpus of spoken ELF was a desirable first target (Seidlhofer, 2001: 146), since spoken language is bound to be more adaptable and open to situational change than written language. An additional goal for ELF corpora was the collection and transcription of naturally-occurring unscripted interactive data, since these are “overtly reciprocal […] allowing for observations regarding the intelligibility of what interlocutors say” (Seidlhofer, 2001: 146). A strong focus on naturally-occurring unscripted interactions has therefore been a key element of ELF research for at least two decades—and also pertains to ELF corpora. Hence, although spoken corpora may be a minority in the big picture of *English corpus linguistics, they are central pillars of ELF research.

The fact that many ELF scholars (e.g. Cogo, 2012, 2020; Pietikäinen, 2014) work with their own data and draw on CA (e.g. Kaur, 2021) when focusing on the study of language-use-in-interaction has, of course, also affected how corpora like VOICE are built. Most ELF corpora tend to include complete speech events rather than, for instance, decontextualized stretches of language with a fixed word count. In a similar fashion, transcripts in ELF corpora (and VOICE in particular) tend to represent conversational interaction with a fairly high amount of detail compared to non-ELF corpora. The *Spoken BNC 2014*, for instance, renders pauses with a broad distinction between “[s]hort pauses (up to five seconds)” and “long pauses (more than five seconds)” (Love, 2020: 116). This representation is clearly very different from CA transcripts where pauses tend to be timed to the tenth of a second (cf. Jefferson, 2004: 25). By comparison, pauses in VOICE transcripts are “timed to the nearest second and marked with the number of seconds in parentheses” (VOICE Project, 2007: 2). In addition, a brief pause of “up to a good half second” (VOICE Project, 2007: 2) is indicated with a separate tag in VOICE transcripts. Taking pauses as an indicator, the representation of spoken language in VOICE is thus not quite as precise as in CA transcripts, but quite detailed.

VOICE transcripts are orthography-based, but contain a comparatively high amount of spoken mark-up. They represent spoken phenomena like repetition, fillers, word fragments and discourse markers and they provide mark-up for conversational phenomena like overlapping speech, laughter, speaking modes (like <soft> or <fast> or laughingly spoken), pronunciation variations and coinages, onomatopoeic noises and also non-*English speech (see VOICE Project, 2007). As will be shown below, having this kind of mark-up available opens up possibilities of combining principles of corpus linguistic and CA methodology when working with the data.

## Data

### Investigating Transient International Groups (TIGs): Comparing Group Constellations

The data analyzed in this paper are interactions in three Transient International Groups recorded in the *Vienna-Oxford International Corpus of English* (*VOICE*), a one-million-word corpus containing 151 spoken ELF interactions in total. The term Transient International Groups (TIGs) refers to groups of multilingual speakers who interact for a particular purpose at a particular location for a certain amount of time and then dissolve again (Pitzl, 2018). The three TIGs investigated in this article were chosen since they involve participants who were predominantly or partly unacquainted when the recorded interactions took place. Two groups, which will be referred to as the MALTA-TIG (LEcon329, LEcon547, LEcon548) and the student-TIG (LEcon560), represent casual conversations, whereas the interaction in the BELF-TIG (PBmtg3) is a professional business meeting.^2^ The groups involve four (MALTA-TIG), five (BELF-TIG) and six (student-TIG) main interactants.

**Fig. 1.**
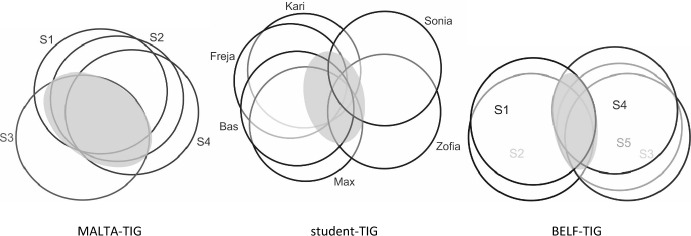
Schematic representation of Multilingual Resource Pool of MALTA-TIG, student-TIG and BELF-TIG (adapted from Pitzl, 2022: 130)

Figure 1 provides a visual representation of the MRP of each group. The three diagrams represent participants’ multilingual repertoires holistically; that is to say, one circle represents one speaker’s entire repertoire.

Crucially, speakers’ repertoires are not limited to their L1s and *English, but are seen to encompass all language—and *languages—speakers ‘know’. This includes all first and so-called foreign or additional *languages as well as any “bits and pieces” (Canagarajah, 2018: 36) of language (or *language(s)) picked up prior to the interaction. If we use the TIGs framework (cf. Pitzl, 2018) to characterize these groups, the MALTA-TIG and the BELF-TIG can be said to feature a *bilateral* MRP constellation, while the six speakers in the student-TIG comprise what we might call a *diverse* MRP. Both constellations can be seen quite often in LF scenarios.

The *bilateral* MRPs in the MALTA-TIG and the BELF-TIG come about as speakers with two different L1s and regio-cultural backgrounds meet and interact using a lingua franca (here: ELF). In the MALTA-TIG, recorded during S3’s visit to Malta, three speakers (S1, S2, S4) know each other very well and have very similar repertoires. ELF use comes about as they interact with S3 who has a considerably different multilingual repertoire.^3^ This situation can be described as an *asymmetric-bilateral* constellation, i.e. a sub-group of three speakers vs. one speaker.

The situation in the BELF-TIG involves two sub-groups of colleagues who work for the same company and have similar multilingual repertoires. These two sub-groups involve two vs. three participants. Nonetheless, each company is represented by one main speaker (S1 and S4) who is supported by one (S2) or two (S3, S5) colleagues respectively.^4^ There is roughly an even balance of 50 % of utterances that are contributed by each sub-group throughout the meeting. Hence, the group constellation in the BELF-TIG can be seen as more or less *symmetric-bilateral* in nature.

The MRP constellation in the student-TIG is somewhat different. The six students share *English as well as some knowledge of *German (and potentially other *languages) in the central area of the MRP but come from six countries. They have six L1s and different regio-cultural backgrounds.^5^ Crucially, they also share no extended interpersonal history or affiliation that precedes the interaction (i.e. there are no sub-groups). This constellation can therefore be described as *diverse* (rather than bilateral).

Both bilateral and diverse MRP constellations can be observed in many LF scenarios and may have an effect on how linguistic and pragmatic conventions emerge in multi-party interactions. Together with the number of participants, the type of MRP is likely to have an effect on how many *languages are potentially available in a group for code-switching. The diagrams in Figure 1 serve to create an awareness of the ways in which speakers’ repertoires overlap in a particular social encounter. The aim of this visualization is to foreground similarities and differences across groups with an emphasis on constellations of resource pools, without, however, emphasizing particular *languages or so-called native/non-native speaker status of participants.

By analyzing transcultural encounters from a TIGs perspective, the multilingual nature and the group dimension of spoken interaction are brought into focus—as is the fleeting nature of many social encounters. Choosing to work with interactive data among predominantly unacquainted international participants, the TIGs approach seeks to provide a new analytic lens that allows us to sharpen our perception of the initial emergence and interactive negotiation of pragmatic conventions. To observe this process of pragmatic norm emergence and negotiation, the present study investigates the use of multilingual elements. A central question in this respect is how multilingual (i.e. non-*English) elements are rendered in the transcribed data and how easily these can be retrieved in the corpus.

### Representation of Multilingual Elements in VOICE Transcripts

Transcripts in VOICE include mark-up that indicates when speakers use words, phrases or utterances that are not *English. In doing so, the mark-up distinguishes between speakers’ L1s, tagged <L1> </L1>, and so-called other *languages, tagged <LN></LN> (VOICE Project, 2007: 4–5). Each L1/LN tag provides information about the *language ‘switched into’ (e.g. *Serbian, *Korean) and a translation into *English where possible. When part-of-speech (POS) tagging was added, all non-*English words were additionally annotated with a POS tag indicating a so-called ‘foreign word’ (FW) (see Osimk-Teasdale 2014; VOICE Project, 2014). With the release of the new open-access online interface for VOICE in 2021, tags for non-*English speech also became searchable in VOICE 3.0 Online (see https://voice3.acdh.oeaw.ac.at) alongside other categories of spoken mark-up (e.g. pauses, overlapping speech, laughter, see Osimk-Teasdale et al., 2021: 14). Queries allow a differentiation between L1 and LN tags and searches for particular *languages and can be carried out for the entire corpus or in customizable sub-corpora, including specific corpus texts.

The analysis in this article has made use of the new VOICE 3.0 Online interface alongside qualitative data coding in MaxQDA to investigate the emergence of multilingual practices. While Section "Use of multilingual elements in three TIGs: A corpus linguistic account" provides a corpus linguistic account of multilingual use in the three groups, Section "The emergence of inclusive vs. exclusive multilingual practices: An in-depth micro-diachronic account" provides a micro-diachronic perspective on the emergence of pragmatic conventions concerning multilingual practices.

## Use of multilingual elements in three TIGs: A corpus linguistic account

If we begin with a quantitative perspective on use of *languages other than *English in the three TIGs, the first general observation is that all three groups include an unusually high number of multilingual elements. Table 1 provides the number and percentage of utterances that include at least one non-*English element (i.e. at least one L1/LN/LQ tag).^6^

**Table 1:** Number of utterances with multilingual elements in the three TIGs

	Number of utterances with multilingual elements (L1/LN/LQ tag)	Total number of utterances	Percentage of utterances with multilingual elements
MALTA-TIG	115	1,464	7.86
Student-TIG	218	3,038	7.18
BELF-TIG	174	4,374	3.98
In total	507	8,876	5.71

Results extracted from VOICE 3.0 Online (https://voice3.acdh.oeaw.ac.at) on 22 August 2023. The numbers given for the MALTA-TIG (in this table and all figures below) reflect the sum of all occurrences in LEcon329 and LEcon547 and all occurrences until utterance 375 in LEcon548. The reason for this cut-off point is that utterance 374 in LEcon548 constitutes a peak in multilingual group development, so to speak, when S3 utters the LN *Italian idiom code-switch *fuma come un turco* (see Pitzl, 2018).

In total, 507 utterances in the three TIGs contain at least one (or more) non-*English words. These 507 utterances make up 15.88 % of all utterances with multilingual elements in the entire VOICE corpus (n=3,192). Note that the high frequency of multilingual elements in the three TIGs does not indicate, however, that multilingual elements are limited to only a few speech events in the corpus. Of the 151 speech events in VOICE, 84.11 % (n=127) include at least one instance of mark-up that indicates non-*English speech.

With close to 4 %, the number of utterances with non-*English speech in the BELF-TIG is substantial. Yet, as shown in Table 1, the percentages in the student-TIG and the MALTA-TIG are even higher, with 7.18 % and 7.86 % respectively. Based on these figures, it seems safe to say that multilingual elements are fairly prominent and frequent in the three TIGs. Crucially, this is not to say that the types of multilingual elements are the same in all three contexts.

Table 2 gives the percentages of L1 vs. LN (and LQ) tags in each TIG and, in doing so, brings to light striking differences. Speakers in the BELF-TIG almost exclusively use elements from their L1 (i.e. 95.53 %), LN tags hardly occur. By comparison, speakers in the MALTA-TIG and the student-TIG use both L1 and LN elements, yet with different preferences. 75.18 % of non-*English tags in the MALTA-TIG indicate L1 use, only one fourth (i.e. 24.82 %) signal LN elements. This ratio is practically reversed in the student-TIG. Here, the majority of multilingual elements (i.e. 77.27 %) indicate LN tags, only 21.90 % are L1 elements. The differences between the groups become even more pronounced if we look at the number and distribution of *languages switched into.

**Table 2:** Comparison of L1 vs. other-*language use in the three TIGs

	MALTA-TIG		Student-TIG		BELF-TIG	
Mark-up indicating L1	103	75.18%	53	21.90%	171	95.53%
Mark-up indicating LN	34	24.82%	187	77.27%	8	4.47%
Mark-up indicating LQ	0		2	0.83%	0	0.00%
Total	137	100.00%	242	100.00%	179	100.00%

**Fig. 2.**
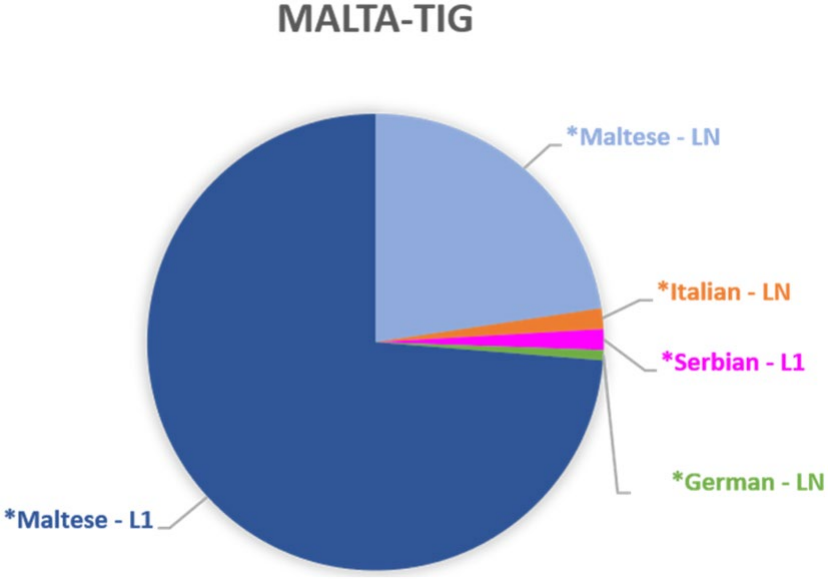
*Languages switched into in the MALTA-TIG (n=137)

As displayed in Figure 2, the vast majority of non-*English tags in the MALTA-TIG indicate use of *Maltese, which include L1 *Maltese (n=101; 73.72 %) and LN *Maltese tags (n=31; 22.63 %). In terms of frequency, *languages other than *Maltese (and *English) only play a minor role in the MALTA-TIG. As will be discussed below (Section "The emergence of inclusive vs. exclusive multilingual practices: An in-depth micro-diachronic account"), this lack of frequency, however, does not mean that other *languages are completely irrelevant. Especially *Italian plays an increasingly important role in course of interaction in the MALTA-TIG, which is, however, not visible on the basis of frequency information alone.

**Fig. 3.**
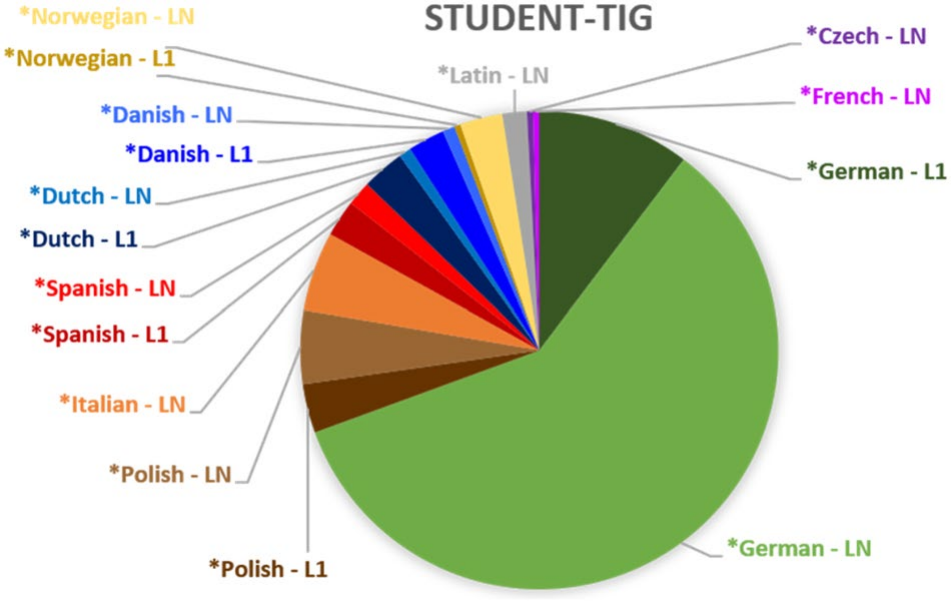
*Languages switched into in the Student-TIG (n=242)

By comparison, the student-TIG (Figure 3) involves a much wider range of *languages. More than two thirds of non-*English elements indicate *German (n=168; 69.42 %), which is an LN for the majority of participants who spend an exchange term in Austria. The remaining 30.71 % of L1/LN tags comprises nine different *languages. These include *Polish, *Spanish, *Dutch, *Danish and *Norwegian (i.e. participants’ L1s), but also isolated use of *Italian, *Czech, *French and even *Latin words. The diverse (rather than bilateral) constellation of the MRP in the student-TIG (as depicted in Figure 1) is thus clearly reflected in the number of *languages used in the group. What is noteworthy in this respect is that all *languages that are L1s of participants occur both as L1 and as LN tags in the transcript. This makes it likely that elements produced by speakers in their L1 are taken up by co-participants for whom they are, of course, non-L1 (i.e. LN) elements. This observation based on frequency can be seen as a first indication of an inclusive orientation to the use of multilingual elements in the group: It is likely that multilingual resources are shared and repeated by speakers in the course of interaction. Yet, more detailed qualitative engagement with the data is needed to substantiate this claim (see Section "The emergence of inclusive vs. exclusive multilingual practices: An in-depth micro-diachronic account").

**Fig. 4.**
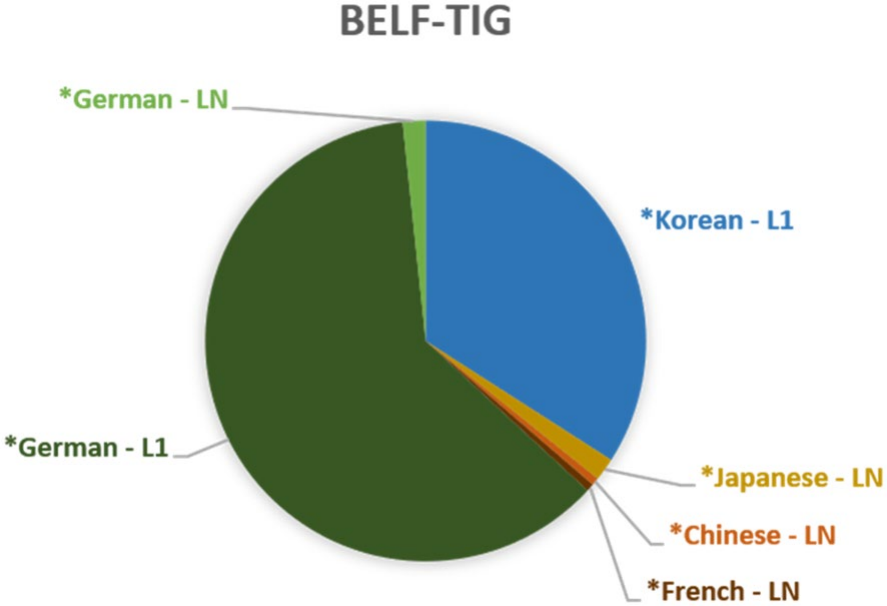
*Languages switched into in the BELF-TIG (n=179)

A very different picture emerges when we look at the *languages used in the BELF-TIG, as shown in Figure 4. In this group, almost all switches (i.e. 95.53%) are into L1. About two thirds (61.45 %) are L1 *German elements, about one third (34.08%) are L1 *Korean. In contrast to the other two TIGs, these frequencies indicate that participants of the BELF-TIG do not repeat their interlocutors’ L1 elements: Repetition and uptake of *German or *Korean words by the other ‘party’ would clearly have led to a higher frequency of LN tags in the data. Based on frequencies, we can tentatively posit that interlocutors in the symmetric-bilateral constellation of the BELF-TIG primarily use multilingual elements exclusively, i.e. within their L1 subgroup, but not with the other ‘party’. Yet, once again, more detailed qualitative engagement with the data is needed to substantiate this claim.

The quantitative corpus linguistic account of mark-up indicating L1/LN use and different *languages provided in this section reveals considerable differences across the three TIGs. The absence of LN elements in the BELF-TIG, for instance, is likely to point to an exclusive orientation in the use of multilingual elements, whereas student-TIG and MALTA-TIG seem more inclusive (L1 and and LN tags). Yet, differences are also observable in the student-TIG and MALTA-TIG, in particular with regard to the number of *languages used. This may be linked to the differences in group constellations, i.e. diverse vs. bilateral MRPs (cf. Section "Investigating Transient International Groups (TIGs): Comparing group constellations").

It is clear that quantitative evidence alone only provides a starting point for these observations, however. In-depth engagement with the data is needed, especially if our interest is in the emergence of group-specific pragmatic conventions. The next section therefore provides a detailed account that draws attention to the micro-diachronic development of pragmatic conventions in interaction.

## The emergence of inclusive vs. exclusive multilingual practices: An in-depth micro-diachronic account

### Observing Inclusive Multilingual Practices: Multilingual Curiosity and Informal Language Learning

The frequencies provided in Section "Use of multilingual elements in three TIGs: A corpus linguistic account" already indicate that participants in both MALTA-TIG and student-TIG seem to use multilingual practices inclusively with their interlocutors, since both L1 and LN elements occur in these groups. To explore this proposition in more detail, we will now turn to a detailed micro-diachronic account of these two groups.

**Fig. 5.**
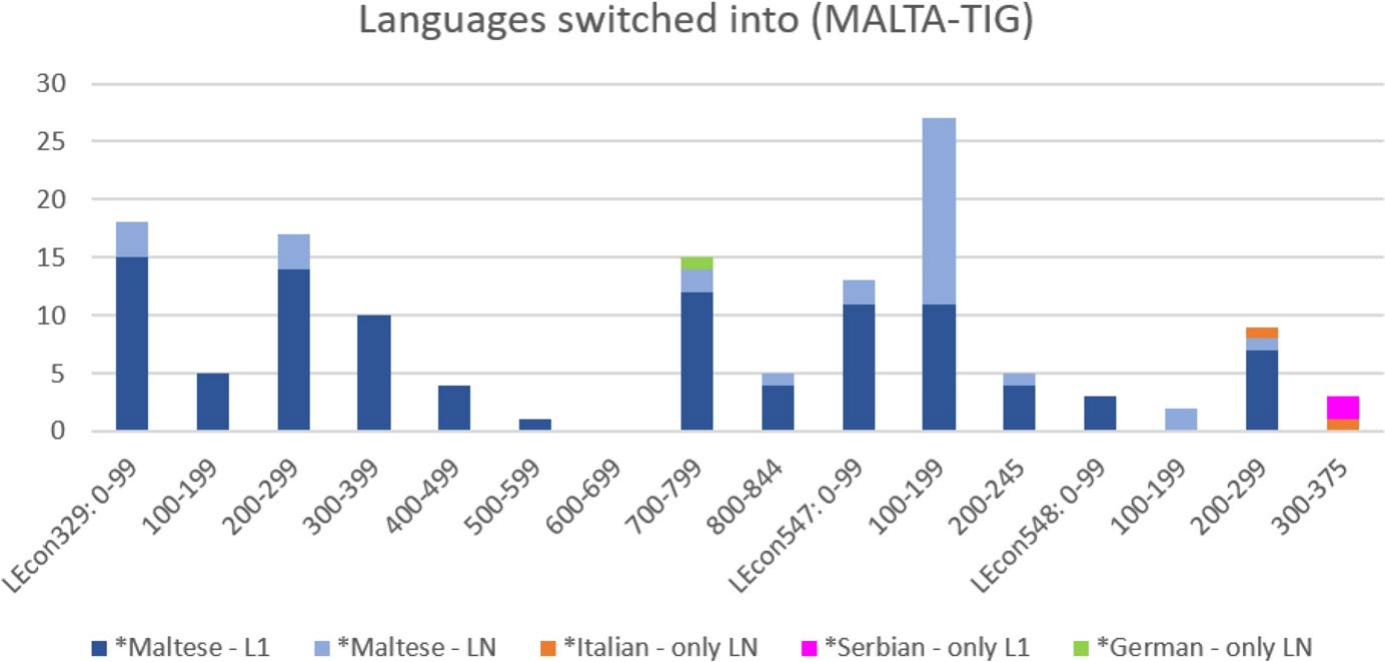
Micro-diachronic view of *languages switched into (MALTA-TIG, n=137; segment length: 100 utterances)

Starting with the MALTA-TIG, Figure 5 maps out the *languages switched into in this group micro-diachronically.

This micro-diachronic representation indicates that the occurrence of *Maltese LN tags becomes more prominent as the conversation progresses. In particular, utterances 100-199 in the second speech event (LEcon547) stand out with a high number of LN and L1 *Maltese tags. This high number is due to what we might call multilingual curiosity and an overt orientation towards informal *language learning exhibited in particular by S3, the L1 *Serbian participant who visits Malta. In utterances 163 and 165, S3 explicitly asks her interlocutors (i.e. the Maltese family she visits) to teach her something in *Maltese. As a consequence of this request, the short exchange in Extract 1 ensues.

Extract 1 (MALTA-TIG; VOICE, 2021: LEcon547)^7^

**Table:** 

168	S4: okay how are you?
169	S3: mhm
170	S4: is <L1mlt> kif inti {how are you} </L1mlt>
171	S3: <LNmlt> kif inti {how are you} </LNmlt>
172	S4: exactly
173	S3: <LNmlt> kif inti {how are you} </LNmlt>
174	S4: <L1mlt> kif inti {how are you} </L1mlt> (.) e:rm what's your name? (.)
175	S3: <LNmlt> kef inti </LNmlt>
176	S4: <L1mlt> kif {how} </L1mlt> (.) exactly
177	S3: not <LNmlt> kif inti kef inti {how are you} </LNmlt>
178	S4: <L1mlt> kif kif inti {how how are you} </L1mlt>
179	S3: <LNmlt> kif inti {how are you} </LNmlt>
180	S4: exactly =
181	S3: =okay (.)
182	S4: a:nd for example you ask me <L1mlt> kif inti {how are you} </L1mlt> and i would say
183	S3: <LNmlt> kif inti {how are you} </LNmlt> (.)
184	S4: i would say <LNmlt> tajjeba {good} </LNmlt>
185	S3: <LNmlt> tajjeba {good} </LNmlt> =
186	S4: =which is <7> good </7>
187	S3: <7> good </7> <LNmlt> tajjeba {good} </LNmlt>
188	S4: very good @@
189	S3: <LNmlt> tajjeba {good} </LNmlt> i know

The exchange clearly shows how enquiring about and informally learning some elements of the local *language (here: *Maltese) is an integral part of building interpersonal relationships in this TIG. While the conversation in Extract 1 takes place with *English functioning as the lingua franca, the density of *Maltese code-switches—and L1/LN tags—in this short stretch is quite remarkable. The interaction clearly shows how the speakers engage in proposing and repeating the *Maltese phrase *kif inti* and an appropriate response (*tajjeba*).

Although *Maltese is clearly important as the main *language of ‘code-switching’ and translanguaging in this TIG, qualitative engagement with the data shows that *Maltese is not the only multilingual and transcultural linguistic reference point that plays a role. In addition to using multilingual elements, speakers negotiate and expand translingual and transcultural territory also by means of metalinguistic discussion. That is to say, interactants do not only switch into but also talk about *languages, countries, regions and regio-cultural specificities.

**Fig. 6.**
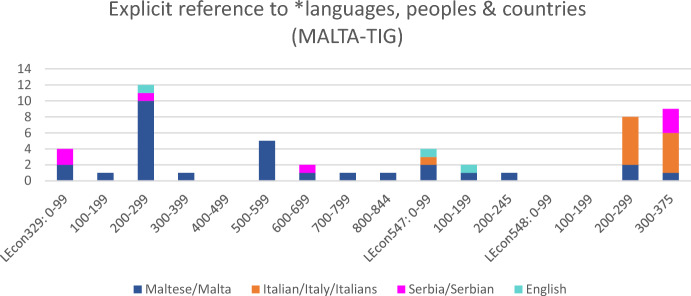
Explicit reference to *languages, peoples and countries (MALTA-TIG, n=51; segment length: 100 utterances)

Figure 6 offers a micro-diachronic perspective on such metalinguistic discussions in the MALTA-TIG by means of tracing instances of the words *Malta/Maltese*, *Serbia/Serbian*, *English* and, crucially, *Italy/Italian(s)* throughout the speech events. While instances of referring to *Malta* or *Maltese* are prominent especially in the first speech event when the speakers are on a sightseeing trip, the diagram clearly indicates that participants also refer to *Serbia/Serbian* and increasingly *Italy/Italian(s)* in the course of their exchange. Especially, utterances 200-375 in the third conversation (i.e. LEcon548) indicate repeated reference to *Italy* and *Italian(s)* in a fairly short amount of time (i.e. just a few minutes). Interestingly, these lexical references to *Italy/Italian(s)* are accompanied by only two code-switches into LN *Italian (cf. Fig. 5).

Extract 2 displays the first of these *Italian code-switches in utterance 251. The excerpt shows how the interlocutors in this TIG jointly become aware of the central role that *Italian plays in their shared MRP, although it is no one’s first language.

Extract 2 (MALTA-TIG; VOICE, 2021: LEcon548)^8^

**Table:** 

251	S3: you say **<LNita> capace?** {able} **</LNita>** {referring to what was on tv} (1)
252	S2: yeah
253	S3: <5> like in italian </5>
254	S2: <5> <L1mlt> kapaci {able} </L1mlt> </5>it's an italian word
255	S3: yes
256	S2: <un> xx xx </un> because (.) as we said last time there are a lot <6> of </6> italian words (.)
257	S3: <6> yes </6>
258	S2: of with italian origin which we were adopted (.) and yes <L1mlt> kapaci {able} </L1mlt> is one of them
259	S3: <LNmlt> kapaci {able} </LNmlt> <7> able </7>
260	S2: <7> yeah </7> (.) <8> able </8> yes
261	S3: <8> able </8>
262	S3: aha (3) {tv can be heard}
263	S2: <L1mlt> jiena jiena {i am} </L1mlt> that's with <L1mlt> jiena {i am} </L1mlt>
264	S3: aha
265	S2: <L1mlt> jiena kapaci {i am able} </L1mlt>
266	S3: a:h okay <1> i'm able </1>
267	S2: <1> <L1mlt> jiena kapaci {i am able} </L1mlt> </1>i'm able i can do it (2) {tv can be heard}
268	S3: because i'm studying italian (.) <2> as well </2>
269	S2: <2> yes </2> (2) but you speak italian <3> don't you? </3>
270	S3: <3> yes i do </3>

Engaging in this metalinguistic exchange about *italian* and *italian words*, the speakers put these *italian words* in relation to *Maltese phrases and words that are rendered as code-switches. In doing so, S3 (i.e. the visitor/guest in Malta) as well as the local hosts (in this passage: S2) demonstrate an overt orientation to language learning—and specifically to learning *Maltese, i.e. the local language. In the course of their interaction, participants increasingly discover that *Italian functions as a common reference point for all of them and is part of their individual multilingual repertoires—and thus part of their shared MRP. In this way, speakers in the MALTA-TIG expand their shared translingual and transcultural territory and pave the way for more advanced inclusive multilingual practices. In particular, their joint metalinguistic work in Extract 2 opens the space for the rare occurrence of an LN idiom code-switch when S3 says *fuma come un turco* in utterance 374. (For a detailed discussion of this idiom see Pitzl, 2018). The MALTA-TIG thus undergoes considerable interactional pragmatic development as speakers successfully expand translingual resources and transcultural territory available to them.

**Fig. 7.**
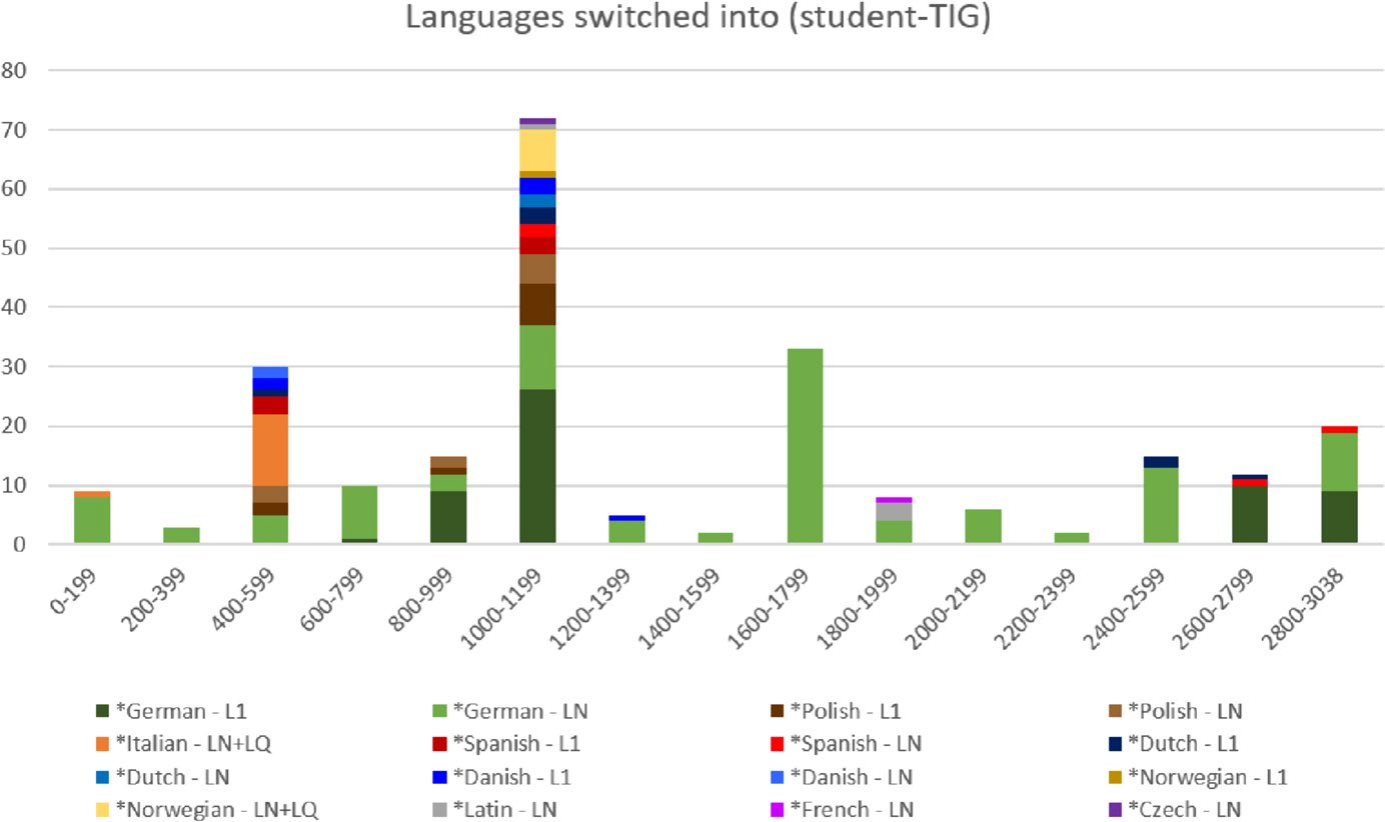
Micro-diachronic view of *languages switched into (student-TIG, n=242; segment length: 200 utterances). Since the conversations in the student-TIG (LEcon560) and the BELF-TIG (PBmtg3) contain more than 3,000 and 4,200 utterances respectively (and are thus longer than the conversations in the MALTA-TIG), the micro-diachronic charts for these two groups (i.e. Fig. 7-10) use of segments of 200 utterances.

Similar to the MALTA-TIG, the student-TIG also exhibits an inclusive orientation towards multilingual use. Figure 7 maps out the occurrence of different L1/LN tags across the two-hour pub conversation micro-diachronically. In addition to the more or less continued presence of LN (and sometimes L1) *German elements throughout the interaction, what stands out in Figure 7 are the high and colorful bars in utterances 400-599 and utterances 1000-1199. Both of these are connected to interactants ‘teaching’ each other to say cheers in different *languages.

The first exchange (utterances 584-599, discussed in Pitzl, 2022) involves *Spanish *chin chin*, *Polish *na zdrowie*, *Dutch *proost*, *German *prost* and *Danish *skål*. The second cheers episode, displayed in Extract 3, features *na zdrowie* and *chin chin*, but also expands the shared MRP with *Norwegian *skal*.

Extract 3 (student-TIG; VOICE, 2021: LEcon560)

**Table:** 

995	SX-m: <soft> <LNpol> na zdrowie (2) na zdrowie {cheers} </LNpol></soft>
996	S1: <yawns>[S3] is learning the same e:r <L1pol> na zdrowie {cheers} </L1pol> (.)
997	SX-f: yeah?
998	S1: yah he's telling her (1)
999	S2: <LNpol> na zdrowie {cheers} </LNpol> (1) {S3 leaves the parallel conversation and joins S1 and S2's conversation}
1000	S3: <1> <LNpol> na zdrowie {cheers} </LNpol> </1>
1001	SX-m: <1> <LNpol> na zdrowie {cheers} </LNpol> </1>
1002	S1: a:h she already knows that (2) yeah because she (has) (.) <LNspa> chin chin {cheers} </LNspa>i'm like (.) <L1pol> na zdrowie {cheers} </L1pol> yeah (3)
1003	S3: <soft> <un> xxx </un> </soft> e:r spanish (.)
1004	S2: <L1spa> chin chin {cheers} </L1spa>
1005	S3: haeh? (.)
1006	S2: <L1spa> chin chin {cheers} </L1spa> (.)
1007	S3: <LNspa> chin <2> chin </2> {cheers} </LNspa>
1008	S2: <L1spa> <2> chin </2> chin {cheers} </L1spa> yeah =
1009	S3: =really? (1)
1010	S2: yeah =
1011	S3: =i've never heard it <soft> <un> xx </un> </soft> (1)
1012	S2: what do y- what do you say? (1)
1013	S3: <L1nor> skal (1) skal {cheers cheers} </L1nor>
1014	S2: <3> <LNnor> skal {cheers} </LNnor> </3>
1015	S1: <3> <LNnor> skal {cheers} </LNnor> </3>
1016	SX-f: <LQnor> skal {cheers} </LQnor>
1017	S3: yeah (.)
1018	S2: <LNnor> skal {cheers} </LNnor> =
1019	S3: =yeah you even <4> did </4> the <spel> ll </spel> (.)
1020	S1: <4> <LNnor> skal {cheers} </LNnor> </4>
1021	S3: @ =
1022	S2: = <LNnor> skal {cheers} </LNnor>
1023	S1: <LNnor> skal {cheers} </LNnor> (.) {parallel conversation between S4, S5, S6 and S7 ends} {parallel conversation between S1, S2 and S3 starts (100)}

The diverse MRP constellation of the student-TIG is clearly reflected in the range of *languages and the density with which these are used in the ‘cheers’ sequences. The enactment of emergent group identity as *multilingual* is, however, not limited to the ‘cheers’ episodes, but also involves further metalinguistic exchange and episodes of informal language learning.

In the exchange that immediately succeeds Extract 3 (utterances 1024–1053), speakers engage in a metalinguistic discussion about how grammatical gender is expressed in the *German determiner and inflectional system. What is noteworthy about this quite sophisticated metalinguistic discussion is that it starts (in utterance 1024) among two students whose L1 is not *German, namely, S4 from Denmark and S5 from the Netherlands. S7 from Austria (with L1 *German) only joins the discussion from utterance 1043 onwards. The conversation about *German grammatical gender triggers further metalinguistic comments, in particular a comparison of meaning relations between *German *maedchen*, *magd* and *Dutch *maagd* (utterances 1064–1085) and, shortly after, of *German *jungfrau* (1092–1105; 1117), *Danish *jomfru* (1108–1116) and *Polish *dziewica* (1129). Once again, the diversity of multilingual resources that is actively drawn upon by speakers is quite remarkable. Having talked about the *Latin origin of *Polish *kolumna* (1139–1155), a third short ‘cheers’ episode occurs, in which *Polish *na zdrowie* is compared with *Czech *na zdravie* (1156-1175).

These conversational episodes in the student-TIG provide clear evidence of multilingual curiosity and multilingual awareness that gradually increase in conversation as the speakers interact. Informal language ‘teaching’ and learning play a central role in the group, which implicitly helps participants create a translingual group identity. In this way, the students show an overt inclusive orientation towards the use of multilingual elements.

**Fig. 8.**
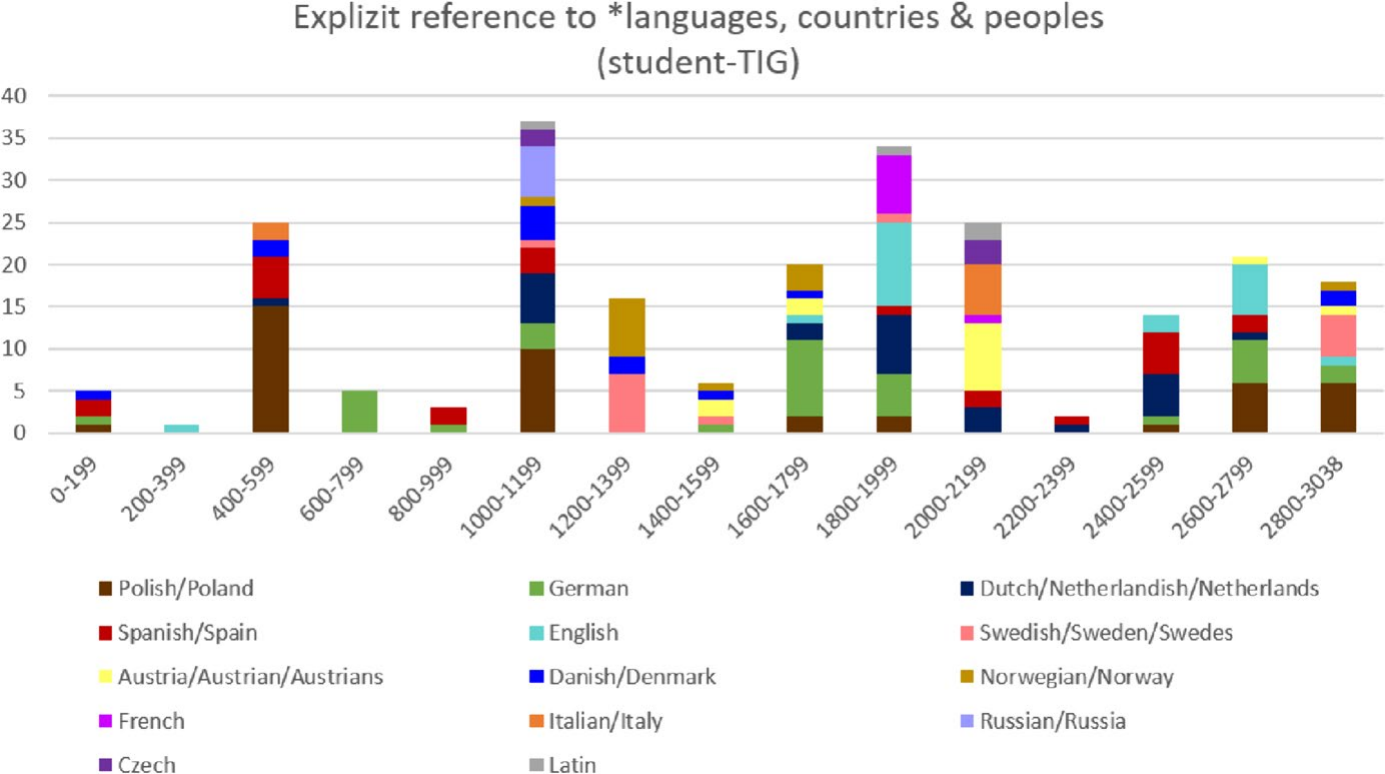
Explicit reference to *languages, countries and peoples in the student-TIG (n=232; segment length: 200 utterances)

Just like in the MALTA-TIG, this ongoing pragmatic process does not only involve using different *language as code-switches; the micro-diachronic development of translanguaging and transcultural identities is accompanied by metalinguistic exchanges that involve referring to *languages, countries and/or peoples in conversation. Figure 8 displays the occurrence of some of these metalinguistic elements micro-diachronically, namely instances of explicit reference to *languages, countries or peoples. The diagram shows that interactants refer to a wide range of *languages, countries and occasionally peoples (e.g. Swedes, Austrians). Especially from the second ‘cheers’ episode (i.e. utterances 995-1023) onwards, the bars in Fig. 8 are increasingly colorful. This is because speakers increasingly compare and relate different *languages and regio-cultures in relatively short stretches of conversation. In doing so, interactants in the student-TIG do not only expand their shared pool of multilingual resources, but continuously also increase their shared awareness of translingual and transcultural similarities and differences.

As demonstrated in this section, both student-TIG and MALTA-TIG exhibit an inclusive orientation towards multilingual use and transcultural awareness. Both groups use non-*English elements and refer to *languages, countries etc. to jointly expand the multilingual and transcultural resources available in the group. Approaching these groups as TIGs, it is noticeable that observable inclusive processes are similar irrespective of the particular *languages and L1 backgrounds represented in a group. Differences revealed by the analysis, for instances with regard to the number *languages switched into, appear to be more of a consequence of different TIG constellations (i.e. bilateral vs. diverse) rather than a consequence of any differences having to do with speakers’ regio-cultural backgrounds.

### Observing Exclusive Multilingual Practices: L1 Side Sequences in a Business Context

In contrast to the MALTA- and student-TIG, the company representatives in the BELF-TIG display a predominantly exclusive orientation to the use of multilingual elements. That is to say, the non-*English speech used in the course of their three-and-a-half-hour interaction is almost exclusively comprised of L1 *German and L1 *Korean switches. There are no LN *Korean switches and practically no LN *German switches, except for utterances 78-79, where the Korean visitors refer to the name of a business fair in Germany (*ambiente*). Although the lack of LN elements indicates that the speakers in this business context are clearly not working towards increasing their shared multilingual resources, it is nonetheless of interest to see if the group undergoes some sort of micro-diachronic development.

**Fig. 9.**
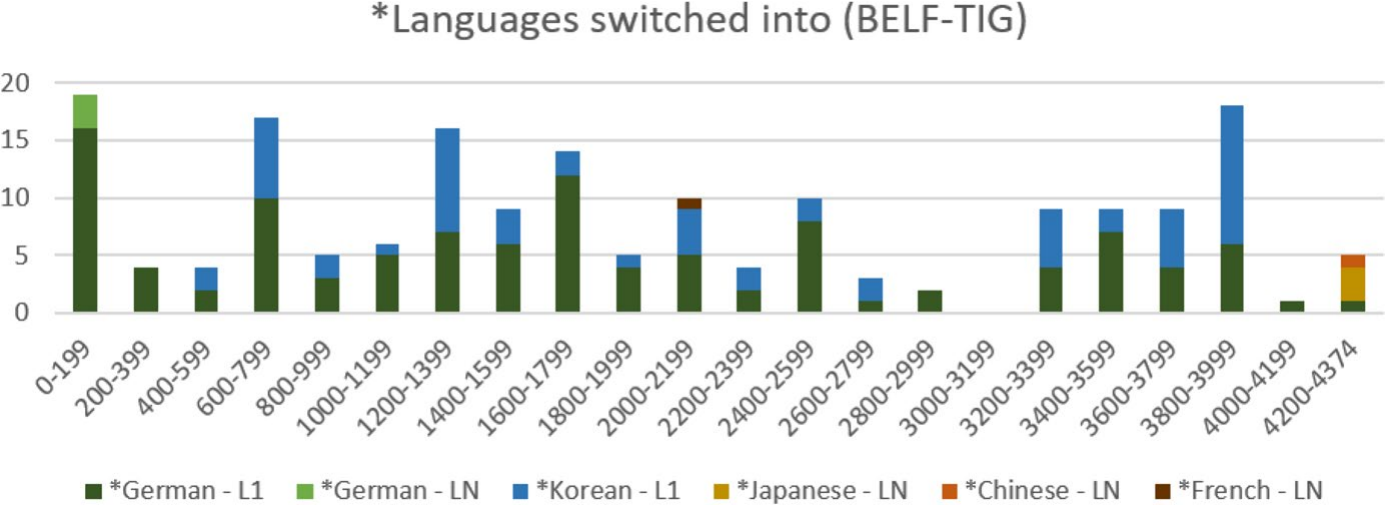
Micro-diachronic view of *languages switched into (BELF-TIG, n=179; segment length: 200 utterances)

Figure 9 shows that switches in the opening phase of the meeting initially involve only L1 *German elements. From utterances 400-599 onwards, L1 *Korean elements also appear in the transcript. As the micro-diachronic chart shows, these remain present, alongside *German, throughout the meeting. With being almost exclusively L1 elements, it is clear that the types of ‘switches’ in Figure 9 are different from the language learning episodes observable in the other two TIGs. As discussed in detail in Pitzl (2021), the Korean and Austrian company representatives of the BELF-TIG primarily use their L1s in short side sequences to clarify details with their colleagues. To illustrate this exclusive multilingual use, Extract 4 renders the first short L1 side sequence triggered by the Korean visitors. Extract 5 shows how both an Austrian (S5) and a Korean (S1) interlocutor briefly switch to their L1 in order to check something with a colleague.

Extract 4 (BELF-TIG; VOICE, 2021: PBmtg3)^9^

**Table:** 

439	S1: no no (.) er sur- surprise (.) kinder surprise no.
440	S4: o<8>kay </8>
441	S1: <8> be</8>cause of (1) <to S2> <soft> <L1kor> xxxx? </L1kor></soft> </to S2> (1)
442	S2: <to S1> <soft> <L1kor> xxxx </L1kor></soft> </to S1> (.)
443	S1: we we e:r actually tried to:: to: to do some activities in this (area) but sav-
444	S4: mhm
445	S2: er the infant (.) safety
446	S1: infant safety <2> issue? </2>

Extract 5 (BELF-TIG; VOICE, 2021: PBmtg3)

**Table:** 

1318	S4: <6> and that's for sure the </6> packaging with the (.) plastic? (.)
1319	S5: i think so (.) <L1ger> xxxxxxxx </L1ger>
1320	S4: but why would we launch this (.) this (.)
1321	S1: <L1kor> xxxxx </L1kor>
1322	S4: this packaging? (3)

In both extracts and throughout the entire meeting, L1 *Korean and L1 *German elements are unintelligible for participants from the other company. Yet, these L1 switches appear to be carried out in the spirit of professional efficiency and increasingly seem to become an accepted multilingual practice—albeit an exclusive one. In contrast to the leisure groups discussed in the previous section, their purpose is certainly *not* to expand shared multilingual resources in the group.

As the meeting progresses, Pitzl (2021) demonstrates in detail how L1 side sequences in the BELF-TIG become longer, more numerous and less transparent in terms of content, also leading to longer passages of L1 *German and/or L1 *Korean use that are not transcribed (and hence not represented by L1/LN tags in Fig. 9). What is crucial concerning interactional development and emergence of pragmatic conventions in the BELF-TIG is that the practice that makes the use of unintelligible L1 side sequences acceptable (i.e. non-face threatening) is not in place when the meeting begins. Most company representatives meet for the first time (i.e. T_0_ in the BELF-TIG). They do not assume that using their unintelligible L1 with a colleague is just acceptable. The acceptability and ‘normalness’ of this pragmatic convention is only jointly developed during the meeting by means of accommodative convergence.

**Fig. 10.**
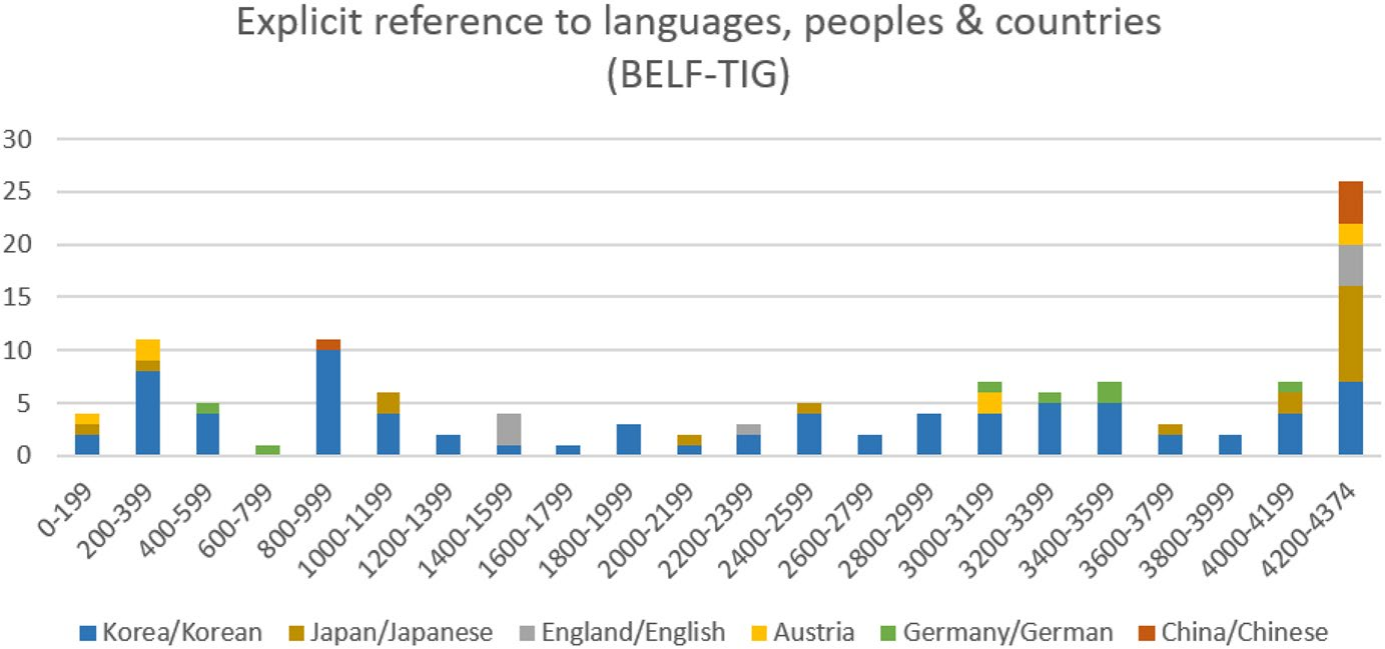
Explicit reference to *languages, peoples and countries in the BELF-TIG (n=122; segment length: 200 utterances)

The fact that emergent multilingual practices in the BELF-TIG are very different from those in the MALTA- and student-TIG is also reflected by the relative absence of metalinguistic exchanges in the meeting. Figure 10 indicates that references to *languages or countries are rather scarce in the BELF-TIG in comparison to the other two groups examined. If anything, Figure 10 shows recurring reference to *Korea* and *Korean* throughout the meeting. Since the main purpose of the meeting is to review and plan distribution and sales of (Austrian) products in Korea, however, these references are prompted directly by the meeting’s content—not by metalinguistic talk. Speakers in the meeting talk about Korean economy (utterance 820), Korean politics (887), the Korean currency won (e.g. 999, 1184, 1430) and the Korean market (2529) for business purposes.

The language *Korean is only discussed at the very end of the meeting during a short small talk phase. After more than three hours, S4 (one of the Austrian hosts) asks whether the *korean language* is *close to any other language* (utterance 4204). This triggers a short metalinguistic exchange in utterances 4205-4261, in which S1 (one of the Korean visitors) explains some characteristics of the *Korean language by referring to Japanese/Japan and Chinese/China as additional reference points. With the exception of this short exchange, metalinguistic talk about *languages does not happen in this BELF context.

The evidence provided in this section clearly shows that multilingual practices in the BELF-TIG are very different from multilingual practices in the student-TIG and the MALTA-TIG. Although there is practically no metalinguistic talk about language or specific *languages in the meeting, the use of unintelligible L1 side sequences involves pragmatic development. Participants appear to converge in the increasing use of this exclusive multilingual practice throughout the meeting.

## Conclusion

This article has examined the use of multilingual elements in three TIGs using data from VOICE. To anchor the micro-diachronic analysis of interaction methodologically, the study has explored the relationship of corpus linguistics and work on spoken interaction. Demonstrating how ELF research and ELF corpora combine both methodologies, the article has discussed multilingual practices as an example of emergent pragmatic conventions and provided details on the bilateral vs. diverse constellations of the MRPs in the three groups.

Adopting a micro-diachronic approach to the analysis of spoken interaction, the empirical study has combined a corpus linguistic (quantitative) as well as a detailed micro-diachronic perspective on the spoken data. The evidence provided has demonstrated that the initial use of non-*English elements as code-switches may evolve towards multilingual practices that can be either inclusive (Malta-TIG, student-TIG) or exclusive (BELF-TIG). In addition to non-*English speech, the presence vs. absence of metalinguistic discussions with reference to *languages, countries and/or peoples also contributes to translanguaging and the creation of shared transcultural territory in groups.

Crucially, both inclusive as well as exclusive multilingual practices are indicative of the emergence of more general pragmatic group conventions. The emergence of such conventions is closely tied to processes like accommodation and the formation of an (implicit) group identity. In the future, it might be interesting to explore how micro-diachronic methodology can be adapted and expanded to describe a broader spectrum of emergent conventions in corpus pragmatics.

## Footnotes

1 In this article, labels for individual *languages (such as *English) as well as the term *language/s are written with an *symbol to convey a post­structuralist understanding that emphasizes non­boundedness and non­homogeneity.

2 LEcon329, LEcon547, LEcon548, LEcon560 and PBmtg3 are identifiers of corpus texts (i.e. speech events) in VOICE.

3 L1 backgrounds in the MALTA-TIG are *Maltese (S1, S2, S4) and *Serbian (S3). For a more detailed discussion of MALTA-TIG (see Pitzl 2018: 39-41).

4 L1 backgrounds in the BELF-TIG are *German (S3, S4, S5) and *Korean (S1, S2). For a more detailed discussion of the BELF-TIG (see Pitzl 2021: 106-109).

5 L1 backgrounds in the student-TIG are *Danish (S4/Freja), *Dutch (S5/Bas), *German (S7/Max), *Norwegian (S3/Kari), *Polish (S1/Zofia) and *Spanish/Catalan (S2/Sonia). See Pitzl (2022: 135) for a more detailed discussion of the student-TIG.

6 An LQ tag is used in VOICE when the distinction between L1 and LN could not be made with certainty (e.g. if a speaker’s L1 is unknown).

7 Excerpts are rendered in the VOICE Transcription Conventions (VOICE Project 2007). Translations within <L1> </L1> and <LN> </LN> are given in curly brackets {}. The symbol (.) indicates a brief pause (i.e. about half a second), number tags like <7> </7> indicate overlapping speech and @-symbols indicate laughter.

8 Bold face has been added to this extract in order to highlight the role of *Italian.

9 Unintelligible speech (e.g. because it is muffled) is represented by xxx in VOICE transcripts.
